# Scurvy: An elusive diagnosis

**DOI:** 10.1002/ccr3.7418

**Published:** 2023-05-28

**Authors:** Michael Pope, Joshua Elder

**Affiliations:** ^1^ Department of Hospital Medicine Piedmont Hospital Augusta Augusta Georgia USA; ^2^ Department of Internal Medicine Augusta University Medical Center Augusta Georgia USA

**Keywords:** ascorbic acid, gingivitis, nutrition, scurvy, vitamin C, vitamin C deficiency

## Abstract

**Key Clinical Message:**

Scurvy is uncommon in the developed world, and clinical presentation may mimic other pathologic states. A thorough social and dietary history is essential to identifying patients at risk of vitamin C deficiency, which can then be easily treated.

**Abstract:**

Scurvy is a disease of defective collagen synthesis that is characterized by easy bruising, gingival hemorrhages, poor wound healing, fatigue, and arthralgias. It is caused by dietary deficiency of vitamin C. This is the case of a patient with multiple risk factors for malnutrition who was diagnosed with scurvy.

## Graphical abstract

Scurvy may present with petechiae, purpura, abnormal hair growth, and poorly healing wounds, as seen in this image of the patient's left arm.

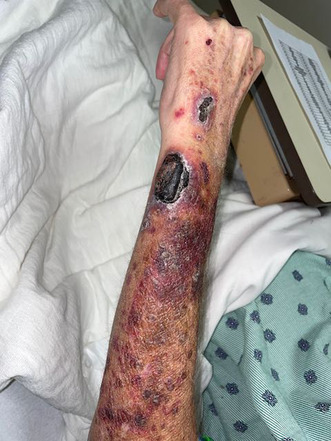

## 1 INTRODUCTION

In 1519, Portuguese explorer Ferdinand Magellan and his fleet departed from Spain, sailing west in attempt to discover a maritime passage to Asia. Following his circumnavigation of South America, the crew spent nearly 4 months crossing the Pacific Ocean, during which time food and water supplies dwindled. Nineteen crew members died of scurvy, and dozens more were made invalid by the time they reached the Philippines.^1^

Scurvy is a disease that primarily affects the connective tissue and is caused by vitamin C deficiency. Vitamin C serves as a reducing agent for the hydroxylation of the amino acids proline and lysine, both essential reactions in the synthesis of collagen. Defective collagen synthesis reduces vascular integrity and results in the classic presentation of easy bruising, petechiae, gingival hemorrhages, and poor wound healing. Additional non‐specific symptoms of scurvy include anemia, fatigue, musculoskeletal pain, joint pain and swelling, and hypotension. Given the differential diagnosis for these signs and symptoms is broad, a dietary history is key to considering and making a diagnosis of scurvy.

Manifestations of vitamin C deficiency have been recorded in ancient Egyptian hieroglyphics,^2^ and have plagued modern populations during times of famine, war, and maritime exploration. In 1753, Scottish naval surgeon James Lind discovered that symptoms of scurvy could be ameliorated by citrus fruits.^3^ Scurvy is now relatively uncommon in the developed world thanks to the convenient availability of vitamin C rich foods. Outbreaks continue to be seen in at‐risk populations, with a recent scurvy epidemic developing in a camp of South Sudanese refugees in Kenya in 2018.^4^

Here we present the case of a 55‐year‐old male who was hospitalized for non‐specific symptoms and found to have poor dietary variety. Scurvy was diagnosed by history and physical exam, and confirmed by serum ascorbic acid concentration. Treatment of scurvy is by supplemental vitamin C, which can bring about full recovery.

## 2 CASE PRESENTATION

A 55‐year‐old male came to the Emergency Department complaining of progressive weakness over the past 2–3 weeks and several pre‐syncopal falls at home. He had no known medical history or consistent primary care. He lived alone and smoked one pack of cigarettes daily and consumed 6–8 cans of beer daily. He used marijuana but no other recreational drugs. His diet had consisted almost exclusively of packaged deli meat, hot dogs, and white bread for the past 3 months. Collateral history from a relative indicated that he declined offers for more balanced food because large meals attenuated the intoxicating effects of his alcohol and tobacco use.

**FIGURE 1:**
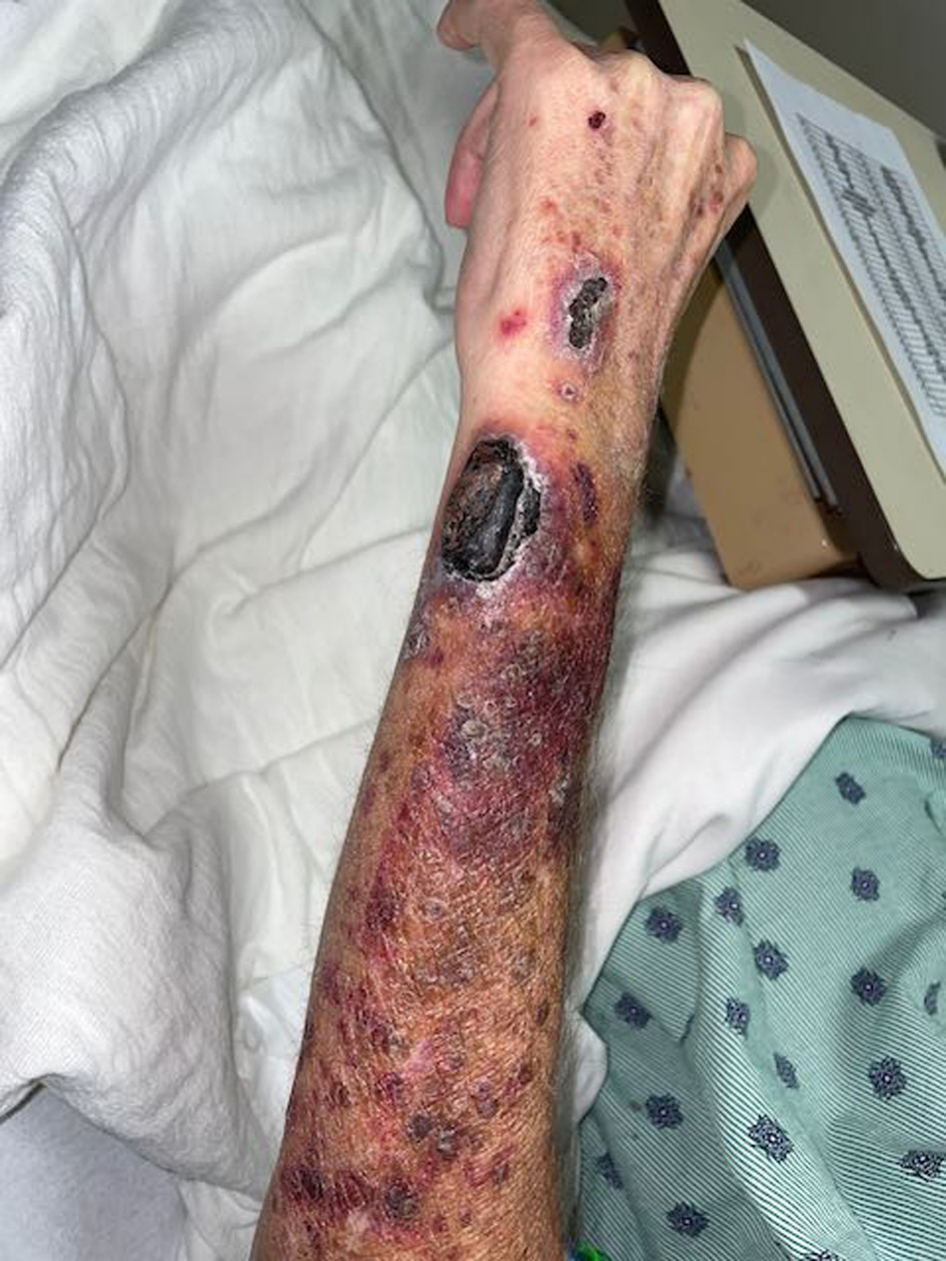
An eschar on the left wrist, which patient reported had been present for several weeks, and surrounding purpura are indicative of collagen dysfunction seen in cases of severe vitamin C deficiency.

**FIGURE 2:**
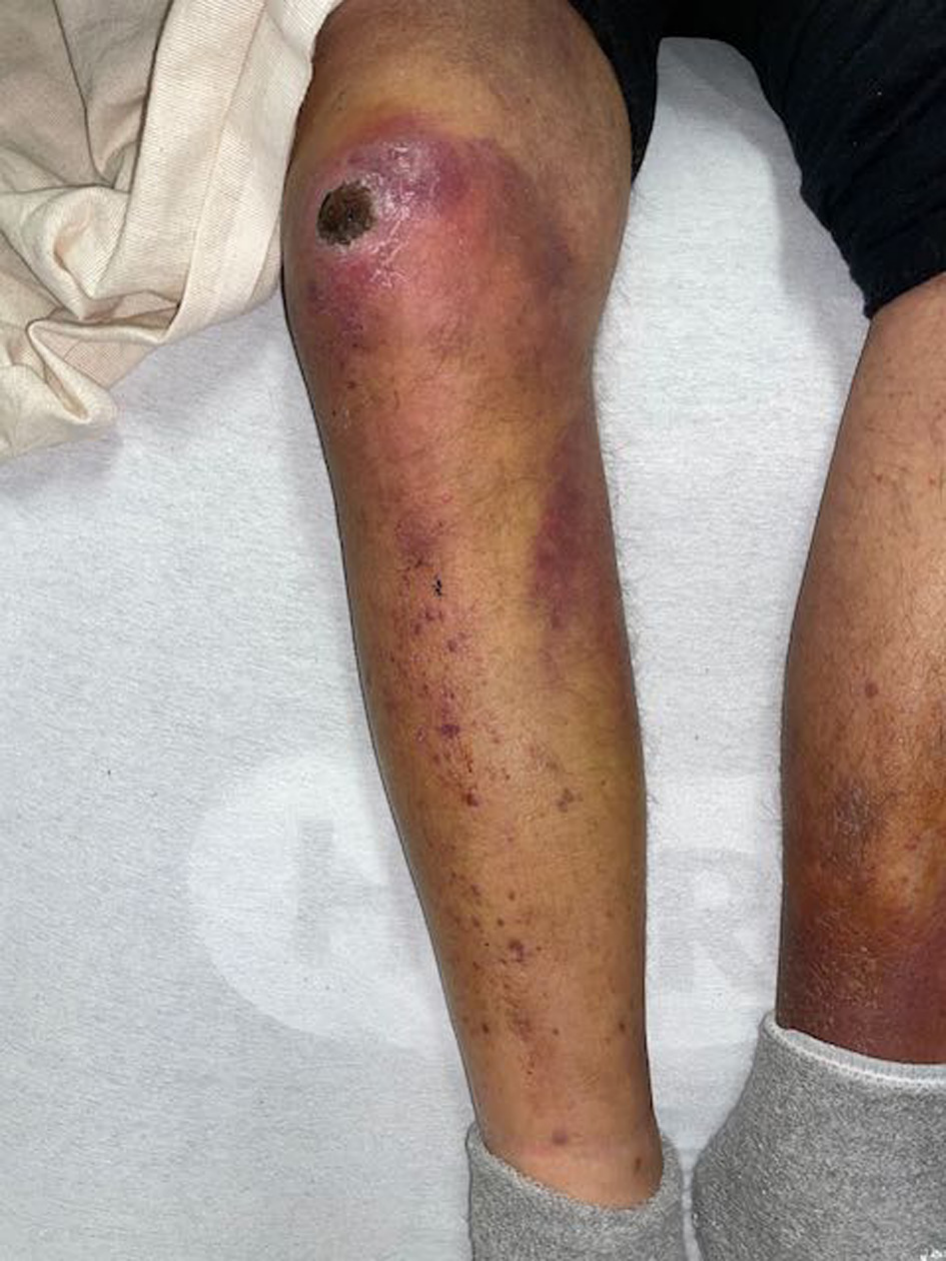
A small eschar, soft tissue edema, and purpura in the lower extremities are indicative of collagen dysfunction seen in cases of severe vitamin C deficiency.

On arrival to the Emergency Department, he was hypotensive with a blood pressure of 84/53 mmHg; all other vital signs were within normal range. He was alert and oriented with normal mental status. He had lost all teeth several years prior; there was no gingival inflammation. There were telangiectasias across the chest and purpura and petechiae on the bilateral upper extremities. There was a large eschar, several centimeters in diameter, on the left wrist which the patient reported had been present for several weeks (Figure 1). The right knee was swollen, erythematous, and also had an eschar (Figure 2); range of motion in this knee was preserved. The bilateral lower extremities had petechiae and pitting edema to the knee. Body hair was kinked.

His hematologic workup revealed pancytopenia (WBC 2.4 thousand/mm^3^, Hgb 6.1 g/dL, platelets 121 thousand/mm^3^) with macrocytosis (MCV 107.5 fL). Serum folate and cobalamin levels were within the reference range. His coagulation profile was notable for prothrombin time 14.8 s (reference 10.1–12.9 s), INR 1.3, PTT 32 s (reference 28–35.7 s). His metabolic profile was significant for a serum sodium level of 130 mEq/L (reference 132–146 mEq/L) and serum BUN 8 mg/dL (reference 9–23 mg/dL). Serum osmolality was 271 mOsm/kg (reference 275–295 mOsm/kg). Renal function was preserved. Liver function tests showed decreased serum albumin of 2.5 g/dL (reference 3.2–4.8 g/dL), mildly elevated AST of 42 U/L (reference 11–35 U/L), normal ALT and alkaline phosphatase (17 U/L and 72 U/L, respectively), and elevated total bilirubin of 3.6 mg/dL (reference 0.3–1.2 mg/dL). Inflammatory markers included a normal ESR (18 mm/h) and elevated C‐reactive protein of 4.62 mg/dL (reference 0.0–0.50 mg/dL).

X‐rays of the left wrist, right knee, and left ankle each demonstrated soft tissue swelling but no acute fractures. There was no radiographic sign of right knee hemarthrosis. A chest X‐ray showed a subacute posterior right seventh rib fracture. A CT scan of the head was without evidence of acute pathology. A CT scan of the abdomen demonstrated heterogenous appearance of the liver parenchyma suggestive of chronic liver disease, and diverticulosis.

He was given 1 L of crystalloid fluid in the Emergency Department and his hypotension resolved. He was admitted to the Internal Medicine service and started on multiple vitamins and a regular diet. He received 1 U of red blood cells for his anemia. A fasting serum ascorbic acid concentration was checked on his second day of hospitalization and resulted as <0.1 mg/dL (reference range: 0.4–2.0 mg/dL). He was started on ascorbic acid 250 mg once daily by mouth. He was discharged home with prescriptions for vitamin C, a daily multivitamin, dietary protein supplements, and home physical therapy.

He was seen for follow‐up in the outpatient clinic after 3 months and was noted to be recovering well. He reported abstinence from alcohol and demonstrated improvement in skin lesions and resolution of pancytopenia.

## 3 DISCUSSION

While the morbidity of scurvy in the general population is unknown, the prevalence of vitamin C deficiency in the United States has been estimated to be 5.9%.^5^ Many clinicians may not frequently consider scurvy on their initial differential diagnosis. A presentation of malaise with bruising, petechiae, gingival swelling, and weight loss may provoke investigation of coagulopathy, hematologic malignancy, or vasculitis before nutritional deficiencies are considered. In this case, the patient and his relative were proactive in disclosing his poor nutritional status. In many cases, though, patients may not volunteer their dietary habits.

Dietary history is not heavily emphasized in clinical training, nor is it a required component of clinical documentation. Nevertheless, the clinician should be aware of risk factors for poor nutritional intake in adults; these include institutionalization, old age, mental illness, loss of teeth, social isolation, and drug and alcohol abuse.^6^, ^7^ In addition to several of these risk factors, this patient also exhibited laboratory parameters consistent with poor dietary solute and protein intake: hypoosmolar hyponatremia, low serum urea nitrogen, and low serum albumin. Interestingly, his serum folate and vitamin B‐12 levels were not diminished, likely because processed meat and bread are well‐fortified with these nutrients. Tobacco use is a risk factor independent of diet that has been associated with low vitamin C levels, thought to be due to increased oxidation of ascorbic acid.^8^

Rapid improvement in scurvy symptoms can be seen within days to weeks of initiating therapy with vitamin C supplementation.^9^ No randomized controlled trials exist comparing the efficacy of different doses of ascorbic acid in patients with scurvy. Recommended doses range from 10 mg daily to 2000 mg daily.^10^, ^11^ It should be noted that the absorption and therefore bioavailability of oral ascorbic acid declines with increasing doses. The bioavailability of 180 mg ascorbic acid is ~90%, but may decrease to <50% for daily doses higher than 1 g.^12^ Furthermore, ascorbic acid is water soluble, and renal excretion increases proportionately to intake to maintain a steady state. A common oral regimen of ascorbic acid for scurvy treatment is 300 mg daily in divided doses for at least 1 month, with careful attention to subsequent daily vitamin C intake. It is unclear whether there is any advantage to higher oral doses of ascorbic acid. Parenteral ascorbic acid preparations are much more expensive than oral preparations and their advantage in patients who can otherwise tolerate an oral preparation is undetermined.

Scurvy is a debilitating but preventable condition that is uncommon in the developed world. The clinical presentation may mimic other pathologic states, obscuring the proper diagnosis. The incidence of scurvy is highly influenced by social factors. By paying careful attention to social and dietary risk factors, clinicians should be able to promptly identify and treat patients with scurvy, leading to full recovery.

## AUTHOR CONTRIBUTIONS

**Michael Pope:** Conceptualization; formal analysis; investigation; writing – original draft. **Joshua Elder:** Supervision; writing – review and editing.

## FUNDING INFORMATION

The authors received no funding for writing this article.

## CONFLICT OF INTEREST STATEMENT

The authors have no conflict of interest to disclose.

## CONSENT

Written informed consent to share de‐identified personal information including photographs was obtained from the subject by the authors.

## Data Availability

The data referenced in this article is available in the case subject's private electronic medical record.
